# Swyer-James syndrome: A cause of adult-onset dyspnea in a patient with adult polycystic kidney disease

**DOI:** 10.1016/j.rmcr.2021.101569

**Published:** 2022-01-03

**Authors:** Ahel El Haj Chehade, Himanshu Bhardwaj

**Affiliations:** University of Oklahoma Health and Sciences Center, Department of Pulmonary, Critical Care and Sleep Medicine, 800 Stanton L Young Boulevard, AAT 8400, Oklahoma City, OK, 73104, USA

**Keywords:** Swyer-james syndrome, Lung hypoplasia, Bronchiectasis, Adult polycystic kidney disease, Dyspnea

## Abstract

Swyer-James syndrome (SJS) is a rare lung condition characterized by abnormal lung growth secondary to childhood post-infectious bronchiolitis obliterans. Usually, one lung is affected more than the other leading to asymmetrical lungs with one lung being significantly smaller. The disease can lead to pulmonary obstructive airflow physiology, bronchiectasis, and fibrosis. Dyspnea usually presents early on in infancy and symptoms can mimic asthma, however, they can go unnoticed until adulthood. We present a case of SJS in a patient with adult polycystic kidney disease (ADPKD) and color vision deficiency. The patient presented to our clinic for evaluation for progressively worsening dyspnea and cough. His imaging revealed a hypoplastic left lung with fibrosis, cystic airway disease, and a small left pulmonary artery. His spirometry revealed an obstructive defect. A Ventilation-Perfusion scan (V/Q) showed a significant reduction of ventilation and perfusion to his left lung confirming the diagnosis of SJS. Both conditions – SJS and ADPKD-are not pathologically or genetically related and are very rare. Having both conditions is even rarer yielding interesting radiological imaging.

## Highlights

• Lung hypoplasia is a rare cause of dyspnea.
• Hallmarks of the disease are loss of lung tissue mass, obstruction on spirometry, fibrosis and occasionally bronchiectasis.
• Treatment varies depending on the severity of the disease.

## 1 Introduction

Swyer-James syndrome (SJS) is an uncommon lung disease with lung hypoplasia as a hallmark [1]. There is more evidence suggesting that the disease is due to post-infectious bronchiolitis obliterans due to repeated childhood lung infections [2,3]. This results in decreased pulmonary vascularity and hypoplasia of the affected lung with the occasional development of bronchiectasis; accompanied by compensatory hyperinflation of the healthy one [4]. This disease is very rare and it is estimated that the prevalence of SJS is around 0.01% of the population [5,6]. Patients usually develop respiratory symptoms in childhood, with chest radiography showing asymmetric lungs and a pulmonary hyperlucency [7]. Patients with SJS leading to bronchiectasis have a worse disease course than patients with bronchiectasis fee SJS [5]. We would like to report a case of SJS syndrome in a patient with adult polycystic kidney disease (ADPKD) presenting with adult-age onset dyspnea and cough.

## 2 Case report

The patient in this report is a 35-year-old male. His past medical history is notable for color vision deficiency, ADPKD diagnosed at age 24 years old due to positive family history and hypertension. He presented to the pulmonary clinic for initial evaluation for gradually worsening shortness of breath and cough for the last few months. He reported his shortness of breath is worse on exertion and was informed by his primary care physician that he could have asthma. He was given an albuterol sulfate rescue inhaler that seemed to provide some relief. His cough was occasional and non-productive. He endorsed no hemoptysis, weight loss, night sweats, or chest pain. He never smoked cigarettes or tobacco products. He never used any illicit drugs and his exposure and environmental exposure history were none contributory. He reported no history of recurrent lung infections and was not aware of any recurrent lung infections in his childhood.

On physical examination, his vital signs were normal including his pulse oximetry. Lung auscultation revealed decreased air entry in the left lung fields with fine scattered rales. No audible cardiac murmurs were noted. Nail clubbing was absent. Palpation for cervical and clavicular lymph nodes was normal. Sinus and oropharyngeal exams were also normal.

**Fig. 1:**
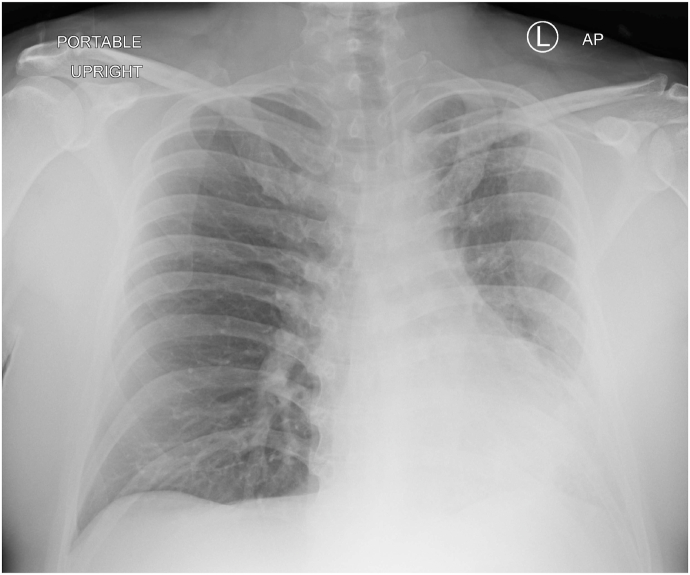
Chest X-ray showing left lung hypoplasia, left lung hyperlucency and reticulations, a hyperinflated right lung, and a left mediastinal shift.

**Fig. 2:**
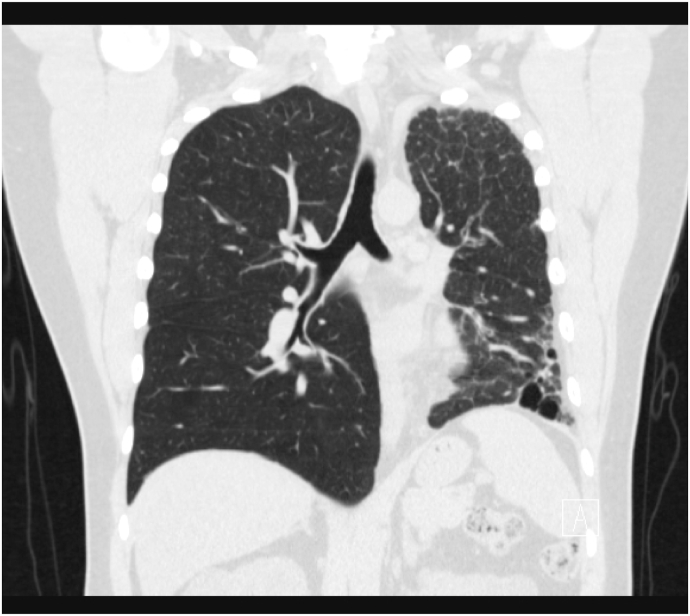
CT scan of the chest showing a hypoplastic left lung with basilar reticulations, pulmonary fibrosis, and cystic airspace disease with a hypoplastic left pulmonary artery.

**Fig. 3:**
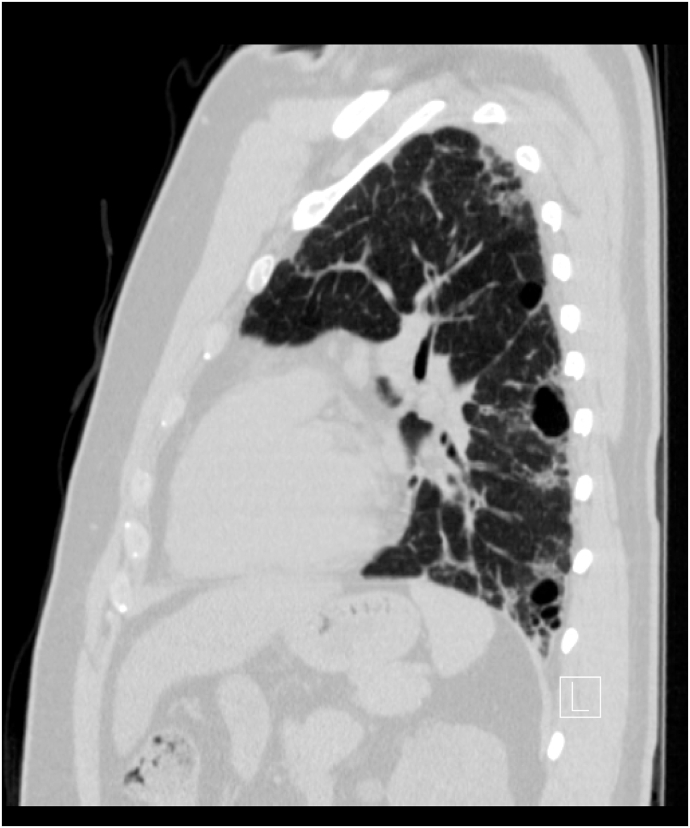
Lateral CT scan view of the chest highlighting left lung volume loss, cystic airspace disease, and fibrosis as well as cystic kidney disease.

**Fig. 4:**
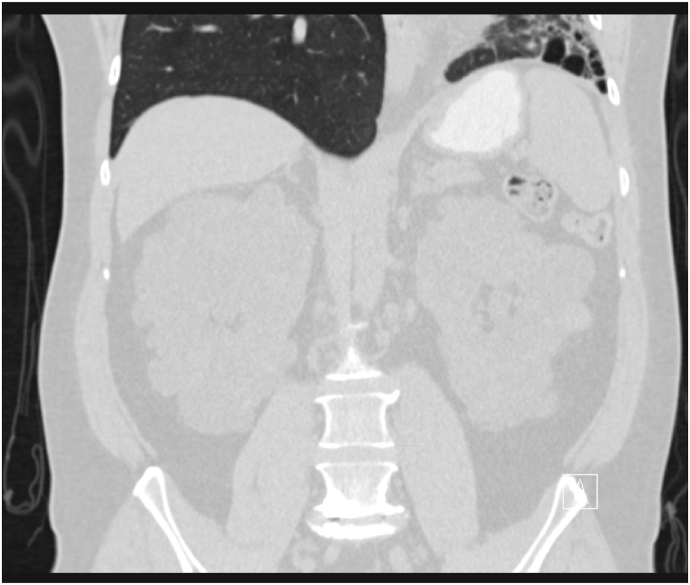
CT scan of the abdomen showing left lung base fibrosis and airspace disease as well as bilateral extensive kidney cystic disease consistent with patient's history of ADPKD.

**Fig. 5:**
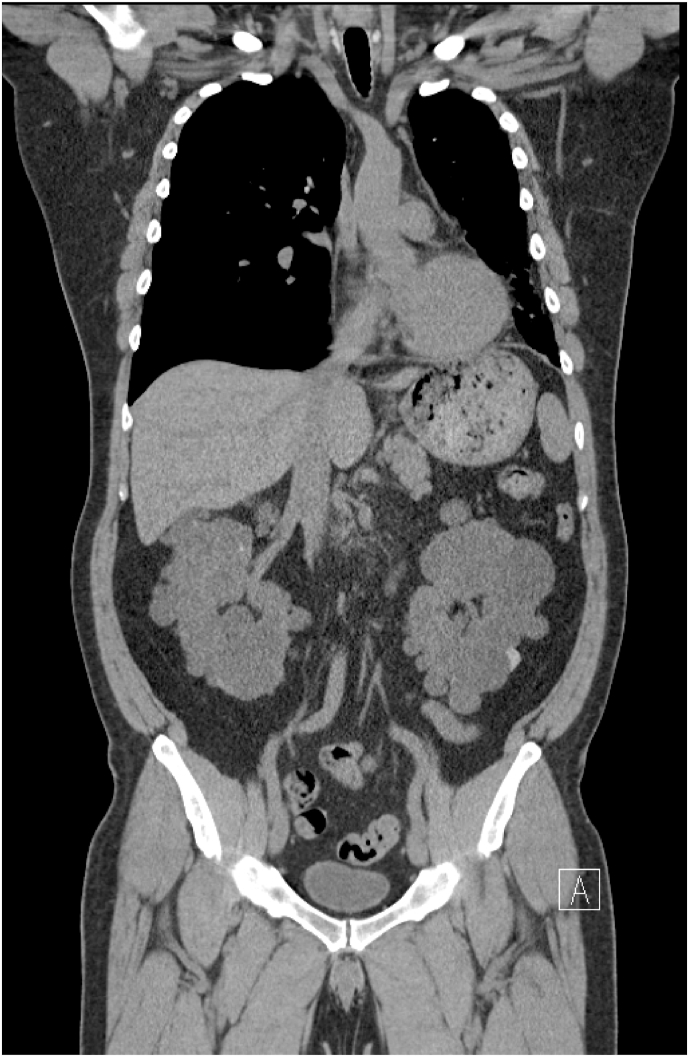
CT scan of the abdomen bilateral extensive kidney cystic disease consistent with patient's history of ADPKD.

**Fig. 6:**
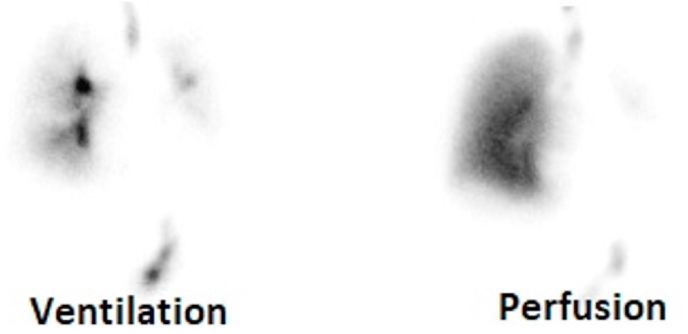
Ventilation-Perfusion (V/Q) Scan showing significantly reduced ventilation and perfusion of the left lung.

A chest radiograph (CXR) revealed left lung volume loss with opacities and reticulations and a left mediastinal shift with a hyperinflated right lung (Fig. 1). A Computed Tomography (CT) scan of his chest showed a hypoplastic left lung with basilar reticulations, pulmonary fibrosis, and cystic airspace disease with a hypoplastic left pulmonary artery (Fig. 2). A later view of the left lung on the CT scan demonstrated better the cystic airspace disease, fibrosis, loss of lung volume, and cystic kidney disease (Fig. 3). An abdominal CT scan interestingly showed the left lung base and the previously described changes as well as the patient's polycystic kidney disease (Fig. 4, Fig. 5). A Ventilation-perfusion (V/Q) scan showed significantly reduced perfusion and ventilation of the left hypoplastic lung (Fig. 6). Spirometry revealed moderated obstruction with a positive bronchodilator response. Lung volumes were at the lower limit of normal with a mild reduction in diffusion capacity for carbon monoxide (DLCO). Results of pulmonary function testing are summarized in Table 1. Further testing revealed no alpha-one-antitrypsin deficiency, cystic fibrosis, or any immune deficiency.

**Table 1:** Summary of pulmonary function testing.

Variable	Value	Lower Limit of Normal (LLN)	Percentage Predicted (%)
FVC in Liters	4.20	4.43	75%
FEV1 in Liters	2.84	3.47	64%
FEV1/FVC	0.62	0.68	–
TLC in Liters	6.83	7.14	88%
DLCO in ml/min/mmHg	27.37	27.65	79%
Flow Volume Loop			

His imaging did not show any bronchiectasis which might be seen in the disease and carries a worse prognosis. The patient was diagnosed with SJS due to his hypoplastic left lung with reversible airway obstruction. The findings were discussed with the patient and he was counseled about his disease. He was started on long-acting bronchodilators along with the rescue inhaler. The patient's symptoms did not resolve completely however he reported mild improvement in his dyspnea and cough after a few months.

## 3 Discussion

SJS is a very rare pulmonary disease with a prevalence of around 0.01% of the total population [5,6]. Most patients report recurrent respiratory infections and dyspnea starting at a young age [2,3]. Our patient did not report a history of childhood lung infections even after discussion with his parents. In rare cases such as with our patient, symptoms can be delayed and the disease might present itself in adulthood.

Typically, chest radiography reveals a hyperlucent hypoplastic lung and occasionally an emphysematous hyperinflated normal lung [1,5,7]. This was noted in our patient CXR (Fig. 1) and warranted further imaging. His CT-scan images (Figs. 2 and 3) showed common features of SJS including asymmetric lungs with hypoplasia, fibrosis, and small caliber hypoplastic pulmonary artery [4,5]. The diagnosis was confirmed pathophysiologically by a V/Q scan (Fig. 5) showing decreased ventilation and perfusion in the hypoplastic lung.

Around 50% of the affected individuals will have evidence of obstructive airway disease on spirometry, bronchiectasis, and fibrosis on imaging, with a minority requiring surgical intervention [4]. Worse prognostic findings are the presence of saccular bronchiectasis and recurrent infections [4]. Both complications are absent in our patient yielding a more favorable diagnosis and a less severe disease burden. He reported minimal impact on his quality of life and exercise tolerance. In addition, his symptoms were well controlled with maintenance and rescue inhalers.

As mentioned prior, SJS is caused by childhood lung infections causing bronchiolitis obliterans leading to hypoplasia and scarring [2]. In contrast, ADPKD is a genetically inherited kidney disease with known transmissibility. Pathophysiologically, SJS and ADPKD are unrelated. There is evidence that patients with ADPKD have a higher prevalence of bronchiectasis but not hypoplastic lungs [8]. Our patient did not have bronchiectasis. Having both conditions together is very rare, and we are not aware of any similar case reported in the literature.

## 4 Conclusion

SJS is an uncommon condition characterized by lung hypoplasia. Symptoms can mimic asthma and may manifest in adulthood. Hallmarks of the disease are mainly airway obstruction, lung opacities on imaging and frequently bronchiectasis. It has been thought to be the result of bronchiolitis obliterans after viral or bacterial infections.

## Funding

This report was not funded by any specific grant from funding agencies in the public, commercial, or not-for-profit sectors.

## Authors contributions

Ahel El Haj Chehade MD participated in reviewing the case, collecting data, drafting the manuscript, reviewing the literature. Himanshu Bhardwaj MD participated in supervising the project, reviewing and editing the manuscript.

## Declaration of competing interest

The authors declare that there is no conflict of interest regarding the publication of this article.
